# COVID-19 Vaccine Acceptance, Hesitancy, and Resistancy among University Students in France

**DOI:** 10.3390/vaccines9060654

**Published:** 2021-06-15

**Authors:** Marie Pierre Tavolacci, Pierre Dechelotte, Joel Ladner

**Affiliations:** 1 Clinical Investigation Center, CHU Rouen, U 1073, Normandie University, F 76000 Rouen, France; 2 Department of Nutrition CHU Rouen, U 1073, Normandie University, F 76000 Rouen, France; Pierre.Dechelotte@chu-rouen.fr; 3 Department of Epidemiology and Health Promotion, CHU Rouen, U 1073, Normandie University, F 76000 Rouen, France; joel.ladner@chu-rouen.fr

**Keywords:** COVID-19, vaccine hesistancy, vaccine resistancy, vaccine acceptance, university student

## Abstract

The objectives were to explore, among university students, the level of COVID-19 vaccine acceptance, hesitancy, and resistancy and to determine the motivations and barriers, and the reasons that may change student vaccination decision making. An online observational cross-sectional study was conducted among students of a French university in January 2021 with questions about the intention to be vaccinated against COVID-19, the motivations and the barriers. The convenience sample included 3089 students, with a mean of age of 20.3 (SD = 1.9). To the question on the intention to vaccinate against the COVID-19, 58.0% of students reported that they would choose to have a vaccination, 17.0% reported that they would not and 25.0% were not sure. The main motivations for vaccine acceptance were “I don′t want to transmit COVID-19 to others”, the main barriers for vaccine resistance or hesitancy were “I prefer to wait until I have more experience with these new vaccines”. Age, female gender, being in first three years of study, studied sciences courses and neither sciences nor healthcare courses of study were significantly associated with a higher risk of vaccine hesitancy or resistancy. Self-estimated knowledge of conventional vaccines and COVID-19 vaccines, and confidence in efficiency and safety of conventional vaccination were associated with a lower risk of vaccine hesitancy or resistancy. It is relevant to disseminate evidence-based interventions to promote COVID-19 vaccine acceptability for college students, especially for the students in neither sciences nor healthcare courses of study, as college students will soon be eligible to receive a COVID-19 vaccine.

## 1. Introduction

The death toll from COVID-19 cases and the failed response have highlighted the importance of an effective vaccine to halt the spread of SARS CoV-2 (COVID-19) [1]. Since-December 2020, several vaccines have been authorized for used in the European Union, and many candidate vaccines are under clinical investigation [2]. Yet, vaccine hesitancy is likely to impair the effectiveness of the rollout the COVID-19 vaccine program [3]. The Centers for Disease Control and the French Prevention and the National Authority for Health [4] have prioritized people at a high risk of acquiring the infection or transmitting the disease, or those with pre-existing medical conditions, and seniors [5]. Young adults, specifically college students, are at risk of being infected with COVID-19 and transmitting the infection to others owing to their sense of invulnerability, and can be a source of transmission to at-risk populations [6,7,8,9] and could be superspreaders [10]. The public acceptance of a new vaccine for COVID-19 developed within a short period remains uncertain despite the forthcoming availability. Lin et al. review declining vaccine acceptance, from more than 70% in March 2020 to less than 50% in October 2020 observing demographic, socioeconomic, and partisan divides [11]. France was one of the countries with the lowest vaccine intention rate among the 15 countries in January 2021 [12]. Acceptance of vaccination is a behavior outcome resulting from a complex decision-making process that can be potentially influenced by a wide range of factors. The concept of ‘vaccine hesitancy’ means to delay accepting or refuse vaccination despite vaccination services being available. There is a continuum between those who accept vaccines without a doubt to complete refusal without a doubt [13]. Betsch et al. developed the “5C” psychological constructs to understand the psychological underpinnings of vaccine uptake [14]: “Confidence” defined as trust in the effectiveness and safety of vaccines, in the reliability and competence of the health services and health professionals, and the motivations of policy-makers who decide on the need of vaccines; “Complacency”, defined as perception of the risks of vaccine-preventable diseases being low; “Constraints”, issues the effect of “physical availability, affordability and willingness to pay, geographical accessibility, and ability to understand (language and health literacy) and appeal of immunization service affect uptake”; “Calculation”, referring to individuals’ engagement in extensive information searching, which is related to perceived risks of vaccination and disease risks; and “Collective responsibility”, the willingness to protect others by one’s own vaccination by means of herd immunity.

Vaccine hesitancy has also steadily increased in more than 90% of countries since 2014 [15]. Several determinants modify vaccination decisions and determine whether a person will refuse, delay, or accept some or all vaccines. Individual decision-making regarding vaccination is complex and involves emotional, cultural, social, spiritual, and political factors as much as cognitive factors [16]. The acceptability of the COVID-19 vaccine in the general population is related to the fear of the virus [17,18]. A U.S. study also showed individual criteria for acceptance of COVID-19 vaccination, such as knowledge of vaccine efficacy, duration of immunity it provides, and trust in political leaders and institutions [19]. In China, perceived risk, concerns over vaccine safety and effectiveness, doctors’ recommendations, and inoculation history were common factors [20]. In the previous H1N1 pandemic, a study showed that the vaccination coverage among university students remained very low in the post-pandemic period and doubts about the safety and effectiveness of the vaccine are key elements in their rejection [21]. Scarce studies have been carried out among students [22] to investigate the COVID-19 vaccine hesitancy, mostly among medical students [23,24]. A high coverage rate is necessary to confer herd immunity needed to flatten the epidemic curve. To create effective strategies to increase COVID-19 vaccination, it is imperative to understand the factors that contribute to COVID-19 vaccine intention and behavior, of college student with potential hesitancy or hesitancy of COVID-19 vaccines. Then the objectives of this study were to explore the level of COVID-19 vaccine acceptance, hesitancy, and resistancy and to determine the motivations and barriers, and the reasons that may change student vaccination decision making, and how this differs according to the university course studied.

## 2. Methods

### 2.1. Study Design and Settings

An observational cross-sectional study was conducted among students of the University of Rouen-Normandy, France from 7 to 31of January 2021 with a convenience sample. The questionnaires were electronically distributed via a mailing list to the almost 30,000 students of Rouen-Normandy University. Volunteer students filled in an anonymous online questionnaire. Students over 25 years of age were excluded from the analysis. The observational study design was approved by the Rouen University Hospital’s Institutional Review Board without mandatory informed consent (E-2021-01).

### 2.2. Questionnaire

Development of the questionnaire was informed by a literature review. The data collected were gender, age, the year of study, and course of study classified in three categories: healthcare (medical, pharmaceutics, first year of healthcare (PASS “Parcours Accés Santé Spécifique”), nurse, midwife sciences, and other healthcare students), sciences (e.g., mathematics, biology), and neither sciences nor healthcare (e.g., law, economic).

The questions are displayed in the Table 1 four questions dealt with conventional vaccination (excluding vaccines for COVID-19) about the efficacy, security, usefulness and estimated knowledge, and vaccine intention—the intention of COVID-19 vaccination when it will be possible—was collected. If the student answered “Yes, absolutely” or “Yes, probably”, they were asked to indicate their motivations (several possible answers): If the student answered “No, probably not”, “No, certainly not”, or “I don’t know”, their reasons were collected. These answers were afterwards classified according the “5C”: confidence, complacency, constraints, calculation, and collective responsibility. We also asked which may affect student vaccination decision making.

### 2.3. Statistical Analysis

**Figure 1:**
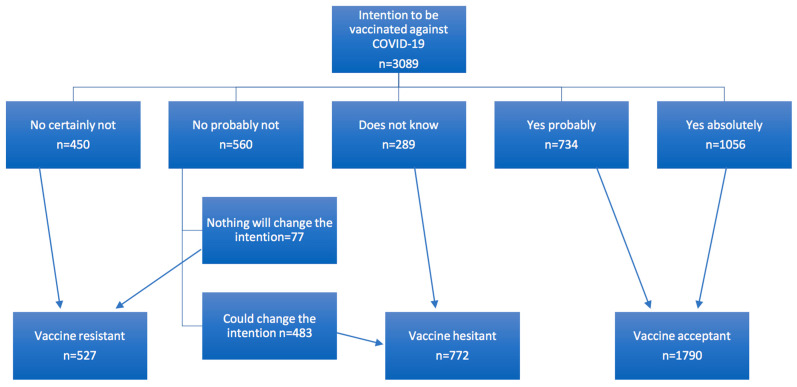
Classification on COVID-19 vaccine acceptant, hesitant and resistant.

A descriptive analysis was performed to define the distribution of the characteristics of the convenience sample. COVID-19 vaccine intention was identified as the dependent variable for the two logistic regression models. Vaccine acceptant (VA) included both those who answered to the COVID-19 vaccine intention question “Yes, absolutely”; “Yes, probably”; Vaccine hesitant (VH) included both those who answered to the COVID-19 vaccine intention question: “No, probably not” and “I don’t know” and vaccine resistant (VR) included the students who answered “No, certainly not” or “No, probably not” and “Nothing, I won′t change my decision” (Figure 1). Categorical variables were described as percentages and 95% confidence interval (CI) and compared using Fisher’s exact test. Continuous variables were described by their mean and Standard Deviation (SD) and were compared using the Student’s *t*-test. Variables with *p* value < 0.20 were included in the multivariate analysis. Logistic regressions were adjusted on age, gender, years of study and courses studied to identified associated factors with VH and VR (VA was the reference).

## 3. Results

A total of 3089 students were included (response rate of 10%) (Table S1), with a mean of age of 20.3 (SD = 1.9), and 71.4% were female. The self-estimated knowledge of conventional vaccines and COVID-19 vaccines was 5.9/10 (2.3) and 4.9/10 (2.3), respectively. Confidence in the efficacy and safety of conventional vaccines (excluding COVID-19 vaccines) was 8.0/10 (2.3) and 7.7/10 (2.3), respectively. The characteristics of the students and factors associated with the COVID-19 vaccine decision are shown in Table 2 and Table 3, respectively.

**Figure 2:**
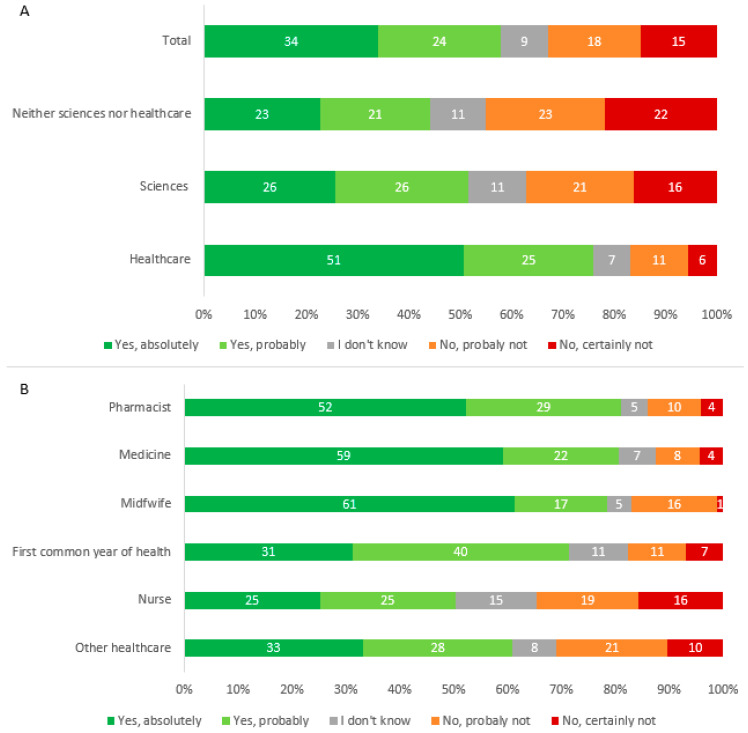
(**A**): Vaccine intention according the courses studied (*N* = 3089). (**B**): Vaccine intention among the healthcare student (*N* = 1999).

**Figure 3:**
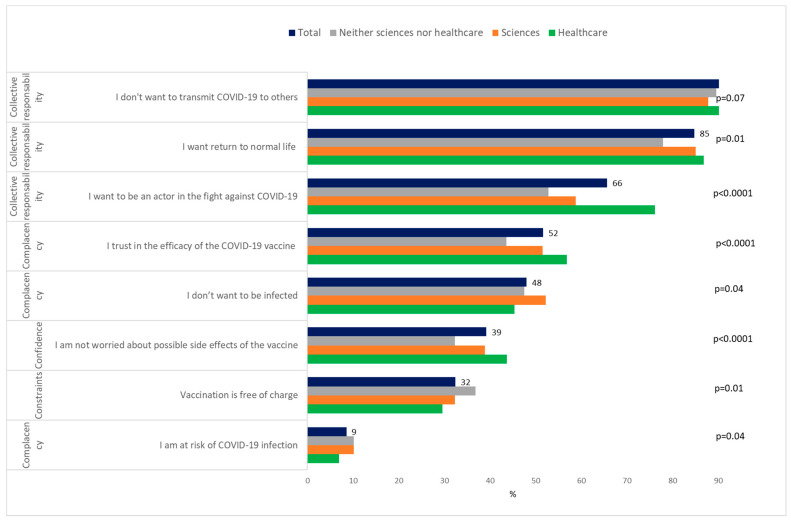
University students’ motivations of COVID-19 vaccine acceptance (*N* = 1790).

**Figure 4:**
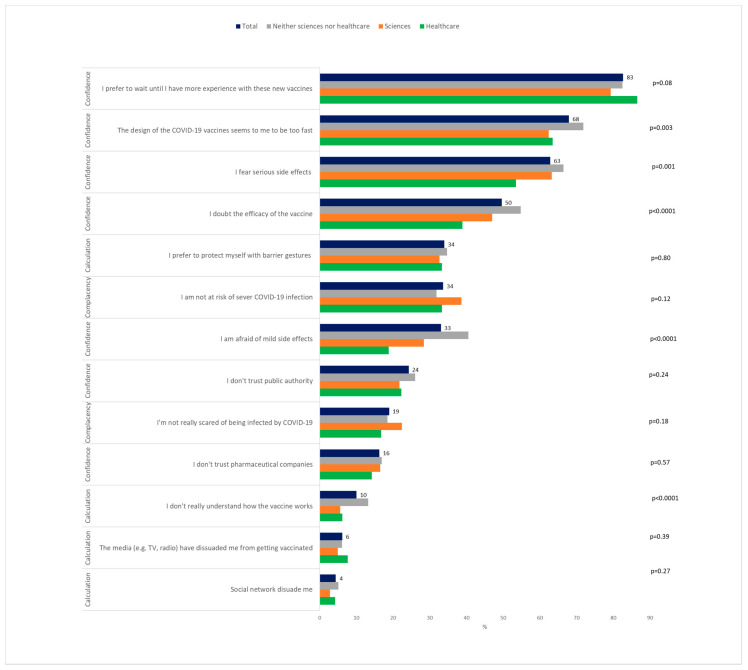
University student’s barriers of COVID-19 vaccine hesitancy or vaccine resistancy (*N* = 1299).

**Figure 5:**
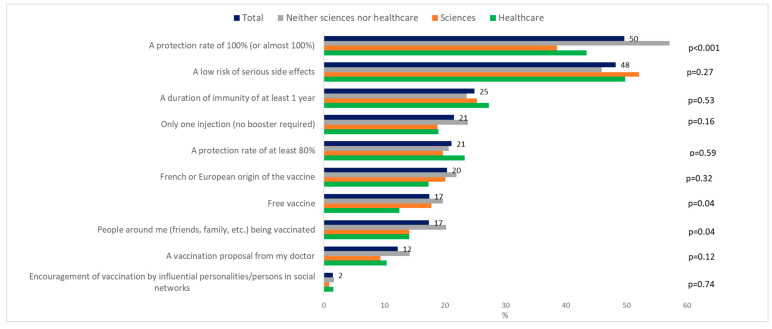
Reasons of change of vaccine hesitant making regarding COVID-19 vaccine (*N* = 772).

To the question on the intention to vaccinate against COVID-19 when it will be possible to do so, 58.0% (1790/3089) answered “Yes, definitely” and “Yes, probably” (classed as VA), 32.7% (1010/3089) answered no (“No, certainly not” and “No, probably not”), and 9.4% (289/3089) did not know. Of the students who answered probably not, 77/560 reported that nothing will change their decision, then 25.0% (772/3089) of students were classed as VH, and 17.0% (527/3089) were VR (Figure 1). Healthcare students were the most likely to want to be vaccinated (75.9%) *p* < 0.0001 (Figure 2A) and among these healthcare students, medical and pharmacy students were the most likely, and nursing students the least likely (Figure 2B) The main motivations for vaccine acceptance were “I don′t want to transmit COVID-19 to others”, “I want return to normal life as soon as possible”, and “I want to be an actor in the fight against COVID-19” (Figure 3). This previous reason was mostly cited by the healthcare students. The main barriers for VH or VR were “I prefer to wait until I have more experience with these new vaccines”, “The design of the COVID-19 vaccines seems to me to be too fast”, and “I fear serious side effects (e.g., hospitalization, serious illness) of the COVID-19 vaccine” (Figure 4). These two last reasons were almost cited by the students of the neither sciences or healthcare courses studied. The three main reasons that may affect student vaccination decision making were “A protection rate of 100% (or almost 100%)”, “A risk of serious side effects that would be rare”, and “A duration of immunity of at least 1 year” (Figure 5). The advice to risk–relatives to be vaccinated against COVID-19 concerned 98.9% of VA, 66.6% of VH and 30.9% of VR (*p* < 0.0001).

Regarding the factors associated with COVID-19 vaccine hesitancy and vaccine resistancy in univariate analysis, differences were identified for each variable, except whether the students had been infected by COVID-19, and whether a relative had been severely infected. In the multivariate logistic regression model, age, female gender, being in first three years of study, studied sciences courses and neither sciences nor healthcare courses of study were significantly associated with a higher risk of VH et VR. Self-estimated knowledge of conventional vaccines and COVID-9 vaccines, and confidence in efficiency and safety of conventional vaccination were associated with a lower risk of VH and VR (Table 3).

## 4. Discussion

To our knowledge, our study is the first one to identify the prevalence and reasons for VA, VH, and VR, as well as the associated factors, among the university student population. Our study shows that, in January 2021, before students have the opportunity to be vaccinated against COVID-19 in France, more than half of the students were VA, a quarter were VH, and one in five students were VR. Vaccination intention is higher than in the general French population (40%) at the same period [12]. The proportion of VA is lower compared to a survey of French students in April 2020 (71%) but the proportion of VR has not changed (19%) [25]. Healthcare students were the most willing to be vaccinated (76%,) similar to the proportion seen in another study [26]. Medical students reported the highest rate of VA, which has also been highlighted among the healthcare workers [16]. Nursing students had the lowest rate of VA (50%) among the healthcare students. As healthcare workers, healthcare students—despite playing a key role in vaccine promotion and patient guidance—are also concerned by the VH hesitancy [27]. In France, a new program of primary prevention interventions among healthcare students, called “Service Sanitaire des Etudiants en Santé” has been shown to improve misconceptions and hesitancy surrounding vaccines [28]. Education about vaccination during medical school in France could be improved with methods based on practical learning methods (case-based learning, clinical placements, and other hands-on methods) [29]. Collective responsibility was the main reasons for VA, and to be actor in the fight against COVID-19 was most cited by the healthcare students, which may be due to motivational and psychological factors, such as the individual′s sense of responsibility for the health of the population and common sense about the value of civic life and social solidarity [30]. The trust in the vaccine’s efficacy and the perceived threat of the COVID-19 only comes afterwards as reasons of VA in contrast to data seen regarding other conventional vaccination where these criteria are paramount [31]. Lack of confidence (speed of the development of the COVID-19 vaccine) were the most common reason for VH and VR as also reported in European general population [32]. Kreps et al. stresses that it is important not to conflate people who are wary of the COVID-19 vaccine and those who are anti-vaccination, as even medically informed individuals may be hesitant because of the speed at which the COVID-19 vaccine was developed [33]. As Palamenghi et al. point out, this mistrust is a factor to be tackled in the battle against COVID-19 [34]. A particularity of young populations is complacency (low perception of disease risk). This finding suggests a need for tailored education messages for college students to emphasize the severity of COVID-19, particularly the potential long-term negative consequences on health, and to address the concerns around side effects of vaccines in general by dispelling misconceptions. Almost a third of VH and VR students prefer to use barrier measures than to be vaccinated, knowing that it is not sure that these protections are actually applied. Indeed, negative vaccine intentions were significantly less likely engaged in the COVID-19 prevention behaviors of wearing masks and social distancing [35]. Having a relative who has been severely infected with COVID-19 does not influence the decision to be vaccinated whereas this factor was found among medical students in a study about measles vaccine [36]. Indeed, the fear of disease does not predominate among students, while fear of COVID-19 is a reason for vaccination in the adult population [37].

Improved efficacy and safety and a reduction in the limitations of the COVID-19 vaccine (duration of immunity of over 1 year) could finally convince students to be vaccinated as also reported in the general French population [38]. In our study, the role of the general practitioner is very weak shifting the opinion of the VH and VR students, while previous studies have shown that receiving health advice about classical vaccination from a doctor or school-endorsed advertisement were predictors of vaccine acceptance [39,40]. The influence of the VA students, as the medical students, should not be neglected with a possible role of ambassador to VH and VR students and to advise their relatives to be vaccinated too.

The very low negative influence of social networks in the choice of the choice not to be vaccinated was reassuring, as the perception of “probable vaccine damage” has been amplified and is easily encountered in the media by those engaged in information seeking, especially from online sources [41]. It may be underreported because students do not want to admit to being influenced by social networks. However, the positive influence of the social networks could be improved by influencers that support the dissemination of scientific insights, including issues related to vaccines and their safety [42].

Women are significantly more at risk of being VH and VR than men as previously found in the general population [17,43]. Courses studied is the factor with the greatest influence on the COVID-19 vaccination intention and with the influence being higher for resistant than for hesitation. Students studying neither sciences nor healthcare courses of study are most at risk of being VH, and especially VR. It would be helpful for these students to increase familiarity with vaccine-preventable diseases, which may lead to improved attitudes towards vaccination among students [44]. It is very reassuring that it is the healthcare students who are the most supportive of the COVID-19 vaccination because, as they can limit nosocomial transmission, and also set an example by encouraging other students and patients to get vaccinated [45]. Regardless of the year and the course of study, knowledge about vaccination and COVID-19 vaccine decreases the risk of VH and VR, a result which has also been seen in healthcare students [26]. Our study shows that knowledge about both conventional and COVID-19 vaccination is moderate and that increased knowledge are equally protective factors against VH and VR. This reinforces the importance of targeting “fake news” to avoid misinformation [46] and increase health literacy [25]. Health literacy could be improved by digital gamification, an innovative option to consider when designing vaccination-related interventions addressed both to the general public and young people in particular, especially for those who are hesitant about vaccination [47]. We also highlight that confidence of efficacy and security in conventional vaccination was a protective factor against VH and VR, which means that it is important that confidence in conventional vaccination improves due to its impact during epidemics. Previously, in the H1N1, accessible information was provided by scientific authorities about vaccine safety, to fill knowledge gaps and address confusion surrounding this issue among students [21]. In our study, having a relative who had been severely ill with COVID-19 did not influence the decision to be vaccinated, whereas this was found to be a factor in a study about vaccine measles [36].

Caution is advised when generalizing these findings, for the following reasons: first, it was a convenience sample in one university in France, voluntary participation could have led to representativeness and self-selection bias in our sample the percentage distribution of our convenience sample did not differ from that of the student population of the University of Rouen-Normandy: 59% of women [48]; second, the proportion of healthcare students was higher (39%) than in that of the University of Rouen–Normandy (15%) but allowed an analysis of COVID-19 vaccination intentions in healthcare subgroups; third the study was carried out just before the start of the vaccination in France and opinions may have changed since that period.

## 5. Conclusions

It is relevant to consider vaccine hesitancy or resistance among university students. This is the right time in the COVID-19 pandemic era to design and disseminate evidence-based interventions to promote COVID-19 vaccine acceptability for college students, especially for the students in neither sciences nor healthcare courses of study, as college students will soon be eligible to receive a COVID-19 vaccine [49]. The quickest factors to implement as advised by WHO to increase COVID19-vaccine acceptance is to adopt three strategies: harnessing social influences (especially medical students could play strong influence as peer student) and increasing motivation (through open and transparent dialogue and communication about uncertainty and risks, including around the safety and benefits of vaccination); creating an enabling environment (making vaccination easy, quick and affordable) [50]. Preventive university medicine, campus-based student organizations, and college students could be consider designing educational programs and messaging that promotes behavioral confidence among college students to receive the COVID-19 vaccine.

## Figures and Tables

**Table 1:** Questions and proposed answers.

Item	Questions	Proposed Answers
Conventional vaccination (excluding COVID-19)		
	Are you confident in the efficacy of the vaccines?	Scale from 0 to 10: 0 “not at all confident” to 10 “very confident”
	Are you confident in the security of the vaccines?	Scale from 0 to 10: 0 “not at all confident” to 10 ”very confident”
	Do you think that getting vaccinated is useful for your health?	Scale from 0 to 10:0 “not at all useful” to 10 “very useful”
	How would you rate your level of knowledge about vaccination?	Scale from 0 to 10: 0 “I know nothing at all” to 10 “I know a lot”
COVID-19 infection		
	Have you been infected with COVID-19?	Yes or no
	Has a relative been hospitalised or died from COVID-19	Yes or no
COVID-19 vaccination		
	Do you intend to be vaccinated against COVID-19 (when it is possible for you to do so)?	«Yes, absolutely”; “Yes, probably”; “No, probably not“; “No, certainly not”; and “I don’t know“
Motivations of vaccination	I am at risk of COVID-19 infection” I don’t want to be infected, I don′t want to transmit COVID-19 to others I trust in the efficacy of the COVID-9 vaccine I am not worried about possible side effects of the COVID-19 vaccine I want to be an actor in the fight against COVID-19 I want return to normal life as soon as possible The vaccination is free of charge	Yes or no
Reasons of hesitation	I am not at risk of sever COVID-19 infection I′m not really scared of being infected by COVID-19 I prefer to wait until I have more experience with these new vaccines I doubt the efficacy of the vaccine I am afraid of mild side effects (e.g., fever, pain at the injection site) of the vaccine I fear serious side effects (e.g., hospitalisation, serious illness) of the vaccine The media (e.g., TV, radio) have dissuaded me from getting vaccinated I don’t trust pharmaceutical companies I don’t trust public authority Social networks (e.g., Facebook, Twitter) have dissuaded me from getting vaccinated I don’t really understand how the vaccine works The design of the COVID-19 vaccines seems to me to be too fast I prefer to protect myself with barrier gestures (e.g., wearing a mask, using hydroalcoholic solution)	Yes or no
Opportunities to change decision	A protection rate of 100% (or almost 100%) A protection rate of at least 80% A duration of immunity of at least 1 year French or European origin of the vaccine A low risk of serious side effects Free vaccine Only one injection (no booster required) A vaccination proposal from my doctor People around me (friends, family, etc.) being vaccinated Encouragement of vaccination by influential personalities/persons in social networks Nothing, I won′t change my decision	Yes or no

**Table 2:** Characteristics of the university students accord the COVID-19 vaccine decision (*N* = 3089).

Variables	COVID-19 Vaccine Acceptance *N* = 1790	COVID-19 Vaccine Hesitancy *N* = 772	COVID-19 Vaccine Resistancy *N* = 527	Total *N* = 3089	*p*
Age mean (SD)	20.5	20.1	20.1	20.3	<0.0001
Women (%)	67.3	78.8	74.8	71.4	<0.0001
Years of study					<0.0001
1 (%)	26.0	41.1	40.2	32.2	
2 and 3 (%)	44.8	42.2	44.8	44.1	
4 and more (%)	29.2	16.7	15.0	23.7	
Courses of study Healthcare (%)	50.8	26.7	15.6	38.8	<0.0001
Sciences (%)	16.9	22.2	21.6	19.0	
Neither Science nor Healthcare (%)	32.3	51.2	62.8	42.2	
COVID-19					
Have been Infected (%)	17.8	15.7	19.0	17.4	0.26
Have a Relative been Hospitalized or Died (%)	15.9	16.3	16.7	16.1	0.90
Knowledge					
Conventional Vaccination Mean (SD)	6.5 (2.1)	5.1 (2.3)	4.8 (2.5)	5.9 (2.3)	<0.0001
COVID-19 Vaccination Mean (SD)	5.5 (2.4)	4.1 (2.3)	4.4 (2.6)	4.9 (2.5)	<0.0001
Confidence about Conventional Vaccination					
Efficacy Mean (SD)	8.9 (1.3)	7.2 (2.1)	5.8 (2.9)	8.0 (2.3)	<0.0001
Security Mean (SD)	8.8 (1.4)	6.8 (2.2)	5.4 (2.8)	7.7 (2.3)	<0.0001

**Table 3:** Factors associated with the COVID-19 vaccine decision (logistic regression *).

Variables	COVID-19 Vaccine Hesitancy ** AOR 95% CI	*p*	COVID-19 Vaccine Resistancy ** AOR 95% CI	*p*
Age	1.08 (1.01–1.16)	0.02	1.08 (1.00–1.17)	0.04
Women	2.09 (1.69–2.57)	<0.0001	1.72 (1.37–2.18)	<0.0001
Years of study				
1	3.08 (2.13–4.44)	<0.0001	3.00 (1.96–4.59)	<0.0001
2 and 3	1.63 (1.22–2.17)	0.001	1.74 (1.25–2.45)	0.001
4 and more	Ref		Ref	
Courses of study Healthcare	Ref		Ref	
Sciences	2.79 (2.17–3.58)	<0.0001	4.50 (3.27–6.19)	<0.0001
Neither sciences nor healthcare	2.92 (2.39–3.59)	<0.0001	6.08 (4.65–7.96)	<0.0001
Knowledge				
Conventional Vaccination	0.81 (0.78–0.85)	<0.0001	0.81 (0.77–0.85)	<0.0001
COVID-19 Vaccination	0.84 (0.81–0.87)	<0.0001	0.90 (0.86–0.94)	<0.0001
Confidence about Conventional Vaccination				
Efficacy	0.61 (0.58–0.65)	<0.0001	0.50 (0.47–0.53)	<0.0001
Security	0.57 (0.54–0.60)	<0.0001	0.46 (0.43–0.49)	<0.0001

* Adjusted on age, gender, years of study and courses studied. ** Reference: COVID-19 vaccine acceptance.

## Data Availability

Data are available on request.

## Supplementary Materials

The following are available online at https://www.mdpi.com/article/10.3390/vaccines9060654/s1, Table S1: Characterics of the university students of the study (*n* = 3089) and of Rouen-Normandie University (*n* = 29,280).
