# Supplementary material for: COVID-19 Vaccine Acceptance, Hesitancy, and Resistancy among University Students in France
Source: Vaccines (Basel). 2021 Jun 15;9(6):654. doi: 10.3390/vaccines9060654 (PMC8232624; doi:10.3390/vaccines9060654)
Supplement: Supplementary file 1 [file vaccines-09-00654-s001.zip › vaccines-1254325-supplementary.pdf]

Supplementary file

Table S1. Characterics of the university students of the study (n=3089) and of Rouen-Normandie University (N=29280)

| | University students of the study (n=3089) | University students o f Rouen-Normandie University (N=29280) |
|---|---|---|
| Women n (%) | 2206 (71.4) | 17275 (59.0) |
| Year of study n (%°) | | |
| 1 | 994 (32.2) | 9370 (32.0) |
| 2 and 3 | 1364 (44.1) | 11 036 (37.6) |
| 4 and more | 731 (23.7) | 8874 (30.3) |
| Course of study n (%°) | | |
| Healthcare | 1197 (38.8) | 4259 (14.5) |
| Sciences | 588 (19.0) | 6098 (20.8) |
| Neither sciences nor healhtcare | 1304 (42.2) | 18 923 (64.7) |
